# Circadian clock disruptions link oxidative stress and systemic inflammation to metabolic syndrome in obstructive sleep apnea patients

**DOI:** 10.3389/fphys.2022.932596

**Published:** 2022-08-29

**Authors:** Xiaoming Li, Xuejian Liu, Qiu Meng, Xinhao Wu, Xin Bing, Na Guo, Xuening Zhao, Xiaozhi Hou, Baowei Wang, Ming Xia, Hui Li

**Affiliations:** ^1^ Department of Otolaryngology, Shandong Provincial Hospital Affiliated to Shandong First Medical University, Jinan, China; ^2^ Department of Thyroid and Breast Surgery, Shandong Provincial Third Hospital, Jinan, China

**Keywords:** obstructive sleep apnea, metabolic syndrome, clock genes, insulin resistance, oxidative stress, inflammatory response

## Abstract

**Objectives:** Obstructive sleep apnea (OSA) is an independent risk factor for metabolic syndrome (MetS). Recent studies have indicated that circadian clock genes were dysregulated in OSA. In addition, it is clear that the impairment of circadian clocks drives the progression of MetS. Therefore, we hypothesized that circadian rhythm disruption links OSA with MetS.

**Methods:** A total of 118 participants, who underwent polysomnography (PSG) and were diagnosed as healthy snorers (control, *n* = 29) or OSA (*n* = 89) patients based on the apnea–hypopnea index (AHI), were enrolled in the present study. General information, anthropometric data, blood biochemical indicators, clock gene expressions, and levels of oxidative and inflammatory indicators were collected, determined, and compared in all the participants.

**Results:** We found that Brain and muscle aryl hydrocarbon receptor nuclear translocator-like protein 1 (Bmal1) and Differentiated embryo chondrocyte 1 (Dec1) were upregulated, while Period 1 (Per1) was reduced in OSA patients. In addition, these changing trends were closely associated with the hypoxia indicator of AHI and have a significant impact on the presence of MetS components, such as hyperglycemia (Dec1 and Per1, *p* < 0.05 and 0.001, respectively), hypertension (Bmal1 and Dec1, *p* < 0.001 and 0.01, respectively), hyperlipidemia (Dec1, *p* < 0.01), and obesity (Dec1, *p* < 0.05). Notably, expressions of Dec1 correlated with IR and predicted the presence of MetS in OSA patients. Finally, we also observed that Dec1 expression was interrelated with levels of both oxidative indicators and inflammatory biomarkers (IL-6) in OSA.

**Conclusion:** This study concluded that circadian clock disruptions, especially Dec1, link OSA with MetS in an oxidative and inflammatory-related manner. Circadian clock Dec1 can be used as a specific biomarker (*p* < 0.001) and therapeutic target in OSA combined with Mets patients.

## Introduction

Obstructive sleep apnea (OSA), characterized by the recurrent complete or partial collapse of the upper airway during sleep, is the most prevalent hypoxia-related disorder worldwide (Benjafield et al., 2019). Chronic intermittent hypoxemia (CIH) is one of the hallmarks of this disease and leads to oxidative stress and inflammatory reaction, which are the potential pathogenesis of endothelial dysfunctions and metabolic disorders (Yamauchi et al., 2005; Gao et al., 2021; Makhout et al., 2021; Maniaci et al., 2021; Ruaro et al., 2021; Cai et al., 2022). Therefore, it is not surprising that OSA has been found to be independently associated with not only cardiovascular or cardiometabolic diseases but also tumors, and neurodegenerative or musculoskeletal disorders (Hirotsu et al., 2018; Benjafield et al., 2019). Among these, metabolic syndrome (MetS) is a clustering of cardiometabolic risk factors that include obesity, dyslipidemia, hyperglycemia, and hypertension. More than half of the OSA combined with MetS and *vice versa* (Hirotsu et al., 2018). Indeed, emerging evidence suggests the complex pathophysiological interactions between OSA and MetS, mainly including insulin resistance (IR) and endothelial dysfunction (Murphy et al., 2017; Hirotsu et al., 2018). Given nocturnal hypoxemia significantly increases the cardiometabolic risks in the population, understanding these physiological perturbations promoted by the OSA is so paramount as that could help to find risk indicators and early therapeutic targets for this common disorder.

Circadian clocks are time- and tissue-specific output genes, which manage the behavioral and molecular processes to synchronize with environmental changes in light and nutrients (Bass and Lazar, 2016). These clock-controlled genes include members of Brain and muscle aryl hydrocarbon receptor nuclear translocator-like protein (BMAL1 and BMAL2), Circadian locomotor output cycles kaput (CLOCK), Cryptochrome (CRY1 and CRY2), Period (PER1-3), Differentiated embryo chondrocyte (DEC), the reverse strand of ERB (REV-ERBα and REV-ERBβ), and kinases (e.g., CSNK1E) (Takahashi, 2017). Recent studies have identified the potential effect of OSA on the disruption of these biological clocks (Burioka et al., 2008; Hou et al., 2019; Yang et al., 2019; Gabryelska et al., 2020; Gaspar et al., 2021). Furthermore, the misalignment of these components is intimately associated with metabolic diseases, such as hypertension, diabetes, obesity, and metabolic syndrome (Yun et al., 2002; Ando et al., 2011; Chen et al., 2015; Chang et al., 2018; Nakashima et al., 2018; Noshiro et al., 2018; Yu et al., 2019; Noshiro et al., 2020; Parameswaran and Ray, 2022). Based on these clues, it is conceivable that OSA-induced disruption of circadian clocks may contribute to its combined metabolic diseases.

Both OSA and MetS are closely associated with oxidative stress and systemic inflammation, which were critical physiological rhythms regulated by the clock genes (Gabryelska et al., 2022). As an independent risk factor for MetS, OSA patients are often in a state of IR and endothelial dysfunction (Stenvers et al., 2019). We speculated that disrupted clock proteins might impact the inflammation and oxidative stress in OSA patients, which in turn facilitated IR and endothelial dysfunction of MetS. However, there is no report regarding the role of circadian clocks in the relationship between OSA and MetS. Therefore, the study aimed to evaluate how biomarkers of oxidative stress and systemic inflammation are linked with clock genes, and further determine whether circadian clock disruptions contribute to the presence of MetS components in OSA patients.

## Materials and methods

### Study subjects

**FIGURE 1:**
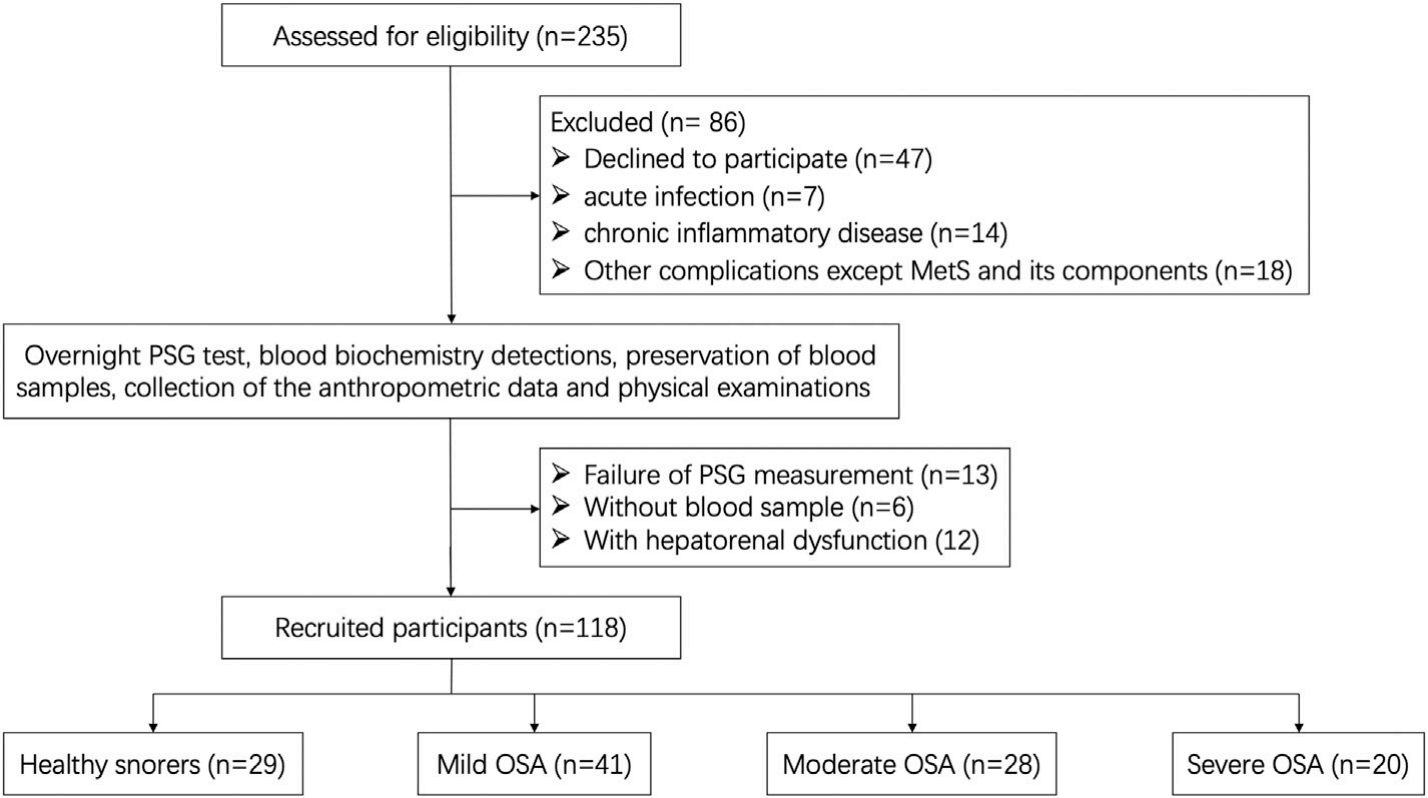
Flowchart of data generation and analysis.

We conducted an observational non-randomized cohort study in Shandong Provincial Hospital Affiliated with Shandong First Medical University. This study was approved by the local ethics committees and was in accordance with the Declaration of Helsinki. All participants provided written informed consent before enrollment. The prevalence of OSA is predominantly male and clock rhythm changes by gender; we therefore, choose a male participant for further investigation. A total of 235 male participants were recruited due to suspected OSA and were recommended for overnight polysomnography (PSG) in the Sleep Center of the Department of Otolaryngology between July 2019 and June 2021. Finally, 117 cases were excluded and 118 cases were selected as eligible participants. A detailed flowchart is shown in Figure 1.

The apnea–hypopnea index (AHI) was defined as the number of apnea and hypopnea events per hour during sleep, according to the overnight PSG results. Based on the severity of the apnea–hypopnea index (AHI), 118 eligible participants were divided into control (healthy snorers, AHI <5 events/h, *n* = 29) and OSA groups (AHI ≥5 events/h, *n* = 89). The OSA patients were further classified into mild (5–14.9 events/h, *n* = 41), moderate (15–29.9 events/h, *n* = 28), and severe (AHI ≥30 events/h, *n* = 20) OSA subgroups.

### Clinical data collection

General information of the participants, such as age and BMI (weight in kg/height^2^ in meter), were collected from the medical records. Anthropometric data, such as systolic blood pressure (SBP) and diastolic blood pressure (DBP), were measured by a digital sphygmomanometer according to a standardized protocol. Blood biochemical indicators, such as glycosylated hemoglobin (HbA1c), fasting blood glucose (FBG), fasting insulin (FIns), homeostasis model assessment of IR (HOMA-IR, FBG in mmol/L × fasting insulin in mU/L/22.5), triglyceride (TG), high-density lipoprotein (HDL), and 12-cytokines (IL-5, INF-α, IL-2, IL-2, IN-6, IL-1β, IL-10, IL-γ, IL-8, IL-17, IL-4, IL-12P70, and TNF-α) were measured by the hospital laboratory department according to routine procedures.

### MetS assessment

According to the diagnostic criteria for MetS by the Chinese Medical Association Diabetes Branch (Lu et al., 2006), the OSA patients were further assigned to OSA without MetS (OSA-non-MetS, *n* = 39) and OSA with MetS (OSA-MetS, *n* = 50) subgroups. In brief, any three of the four following risk factors were identified as MetS: 1) BMI ≥25; 2) FPG ≥6.1 mmol/L, and (or) 2-hour postprandial blood glucose ≥7.8 mmol/L; and (or) validated diabetes; and/or undergoing hypoglycemic therapy; 3) SBP/DBP ≥130/85 mmHg, and (or) confirmed hypertension or in those on anti-hypertensive treatment; and 4) fasting TG ≥ 1.7 mmol/L and (or) high-density lipoprotein cholesterol (HDL-C) <1.04 mmol/L.

### Blood sample processing

For evaluating expression levels of the clock genes and estimation of the oxidative stress status, fasting blood samples (15–20 ml) were obtained from the antecubital vein of the OSA or control patients, and collected in a vacutainer (EDTA tube) at 7:00 a.m. after overnight PSG. Then, blood tubes were sampled by centrifugation (800 g, 20 min, at room temperature). First, the plasma samples were obtained and stored at −80°C until analysis for oxidative detection. Next, peripheral blood mononuclear cells (PBMCs) were collected from the interface bands and subsampled into a clean falcon tube, stored at −80°C for further assays, and thawed at 4°C prior to use, as reported in a previous study (Gaspar et al., 2021).

### Assessment of clock gene expressions by RT-PCR

The total RNA of PBMCs was extracted and purified as recommended by the instructions of the Trizol reagent (Invitrogen). Relative mRNA expressions of the clock genes, such as Bmal1, Clock, Cry1, Cry2, CSNK1E, Dec1, NR1D1, Per1, Per2, and Per3, were evaluated by the real-time quantitative reverse transcriptase polymerase chain (qRT-PCR) as reported previously (Xiao et al., 2019). Primer sequences obtained from Sangon Biotech Co., (Shanghai, China) for the qRT-PCR amplification are listed in Supplementary Table S1. Evaluation of the relative mRNA expressions was performed by the 2^−△△CT^ method (Teng et al., 2012). Fold change of relative mRNA levels was calculated as the average fold change of the OSA samples compared to that of the control samples. The experiments were repeated three times.

### Oxidative stress measurements

Oxidative stress index was evaluated by the detection of the total antioxidant status (TAS), total oxidative status (TOS), and oxidative stress index (OSI) in plasma samples from all subjects. Measurements of TAS and TOS were performed by the standard colorimetric and spectrophotometric methods, using a commercially available diagnostic ImAnOx (TAS/TAC) Kit (Immundiagnostik, Bensheim, Germany). OSI was calculated as a ratio of TOS to TAS.

### Statistical analysis

Statistical analyses were performed using Graph Pad Prism 6.00 (GraphPad Software, Inc.,). Data were shown as the mean ± standard deviation (SD) and analyzed using Student’s *t*-test. Normal distribution of data was determined by the Kolmogorov–Smirnov test. Variables not normally distributed were logarithmically transformed prior to analyses. Correlations of clock gene expressions with AHI, inflammatory markers, or oxidative indicators were assessed by Pearson’s correlation analysis. Based on expression levels of clock genes, receiver-operating characteristic (ROC) curves were calculated to predict the presence of MetS or IR in OSA patients. An area under curve (AUC) of 0.5 represents no predictive power, whereas an AUC of 1 represents perfect diagnostic accuracy. *p*-value < 0.05 was considered as statistically significant.

## Results

### OSA increases the presence of MetS components

A total of 89 OSA patients (39 cases of OSA-non-MetS and 50 cases of OSA-MetS) and 29 healthy snorers (control) were included in the present study, according to the PSG results and diagnostic criteria for MetS. Characteristics of the anthropometric, biochemical, and metabolic data are shown and compared in Table 1. No significant differences were observed in age distribution between the subgroups. As expected, AHI, mean SaO_2_%, and monitoring time spent with oxygen saturation <90% were significantly increased in the OSA-MetS group compared with the OSA-non-MetS group. In addition, BMI, SBP, DBP, HbA1c (%), FPG, FIns, HOMA-IR, TG, and HDL-C in the OSA-MetS groups were much higher than those of the OSA-non-MetS group. Notably, clinical parameters, such as BMI, HbA1c (%), FIns, and HOMA-IR in the OSA group, were higher than those of the control group. Other clinical parameters showed no significant difference between these subgroups. Collectively, these data revealed that a patient with OSA has more frequently MetS components.

**TABLE 1:** Clinical characteristics in control and OSA participants.

Variable	Control	OSA		*P* value^a^	*P* value^b^
		Non-MetS	MetS		
Age (years)	45.38 ± 5.39	45.87 ± 5.17	45.38 ± 4.49	0.837	0.631
BMI (Kg/m^2^)	30.29 ± 5.29	29.05 ± 4.89	31.35 ± 4.61	0.955	**0.025**
SBP (mmHg)	122.62 ± 16.20	121.15 ± 12.17	136.20 ± 12.78	**0.031**	**<0.001**
DBP (mmHg)	75.97 ± 14.21	73.38 ± 8.62	84.62 ± 10.39	0.147	**<0.001**
HbA1c (%)	5.17 ± 1.01	5.22 ± 0.47	6.25 ± 1.53	**0.019**	**<0.001**
FPG (mmol/L)	5.44 ± 1.12	4.92 ± 0.47	5.96 ± 1.26	0.79	**<0.001**
FIns (mU/l)	7.46 ± 2.17	7.56 ± 4.24	13.67 ± 3.71	**<0.001**	**<0.001**
HOMA-IR	1.81 ± 0.71	1.66 ± 0.94	3.58 ± 1.10	**0.001**	**<0.001**
TG (mmol/L)	1.53 ± 0.51	1.10 ± 0.39	2.14 ± 0.73	0.315	**<0.001**
HDL-C (mmol/L)	1.21 ± 0.45	1.42 ± 0.27	1.16 ± 0.36	0.426	**0.001**
AHI (events/h)	2.28 ± 0.85	18.86 ± 17.23	29.27 ± 23.61	**<0.0001**	**<0.0001**
Mean SaO_2_ %	94.46 ± 2.40	90.45 ± 4.84	84.72 ± 7.73	**<0.0001**	**<0.0001**
Time SaO_2_ <90%	7.19 ± 2.52	16.67 ± 13.27	49.73 ± 19.29	**<0.0001**	**<0.0001**

These bold values mean *p* < 0.05. Data are shown as mean ± SD (standard deviation).

^a^ ^/^ ^b^ *p* values are estimated between groups.

a of control and OSA, or subgroups.

b of OSA-non-MetS and OSA-MetS.

BMI, body mass index; fat mass, percentage of body fat; SBP, systolic blood pressure; DBP, diastolic blood pressure; HbA1c, glycosylated haemoglobin; FPG, fasting plasma glucose; FIns, fasting insulin; HOMA-IR, homeostasis model assessment of insulin resistance; TG, triglycerides; HDL-C, high-density lipoprotein cholesterol; AHI, apnea–hypopnea index; AI, arousal index; ODI, oxygen desaturation index; time SaO2 <90%, monitoring time spent with oxygen saturation <90%.

### OSA dysregulated the circadian clocks

**FIGURE 2:**
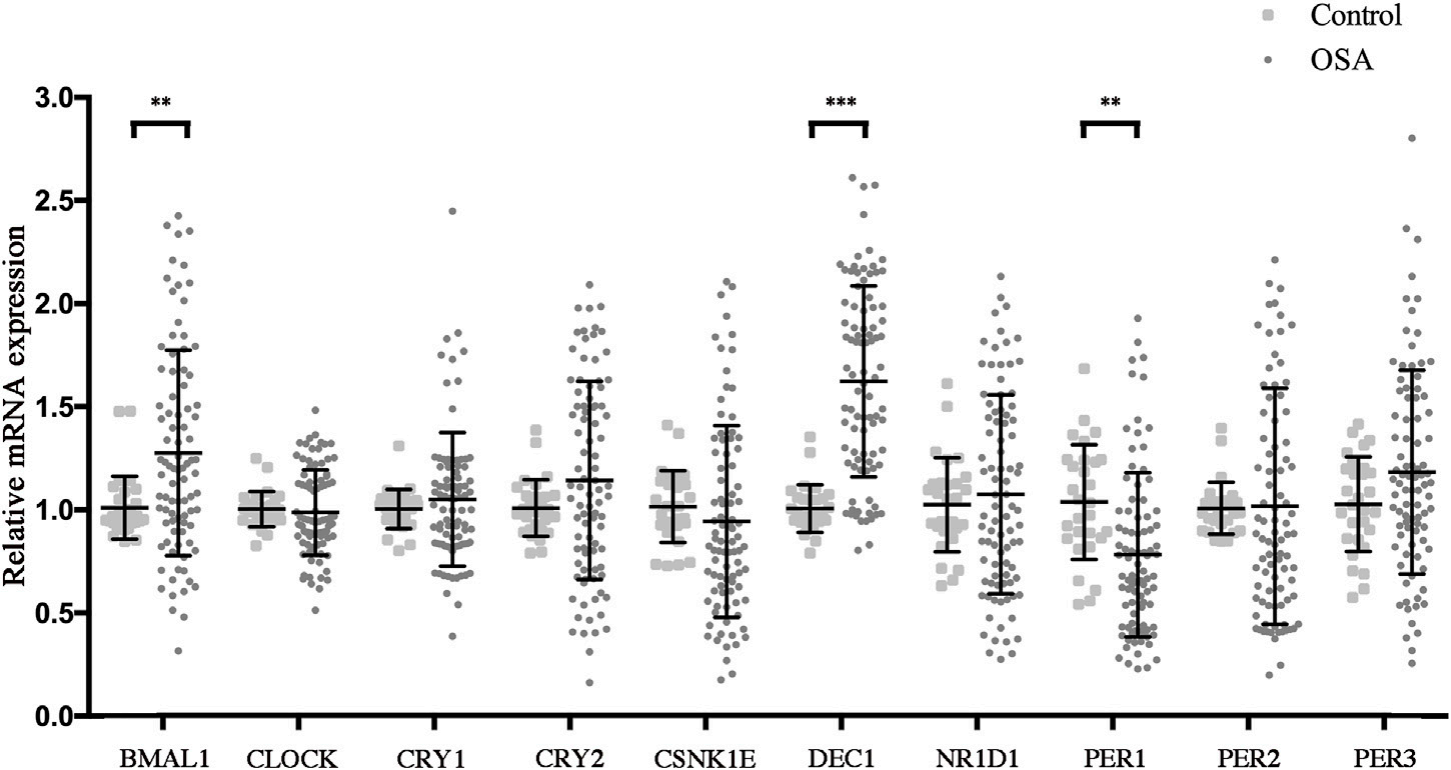
OSA dysregulated the circadian clocks. Relative mRNA expressions of circadian clock genes in the peripheral blood mononuclear cells from control and OSA patients. Mean values from the control group were set at 1.0. Experiments were repeated three independent times. **, *p* < 0.01; ***, *p* < 0.001.

Circadian clock disruptions had been considered as a hallmark of OSA and are reported to play a key role in regulating the pathogenesis of MetS (Burioka et al., 2008; Bass and Lazar, 2016; Yang et al., 2019; Gabryelska et al., 2020; Gaspar et al., 2021). We speculated that some of the major clock alterations may be the key link between OSA and MetS. Therefore, we assessed concentrations of the clock outputs in PBMCs by RT-PCR in the study cohort and further investigated the effect of OSA on the biological clock. The results are shown in Figure 2. Compared with the control group, levels of Baml1 and Dec1 were significantly increased, while that of Per1 was markedly decreased in the OSA group. However, no significant differences were observed for expression levels of Clock, Cry1, Cry2, CSNK1E, NR1D1, Per2, and Per3 between the control and OSA groups. These results indicated that the expression of Bmal1, Dec1, and Per1 in PBMCs is altered in OSA patients.

### CIH is associated with the disrupted BMAL1, Dec1, and Per1 in OSA

**FIGURE 3:**
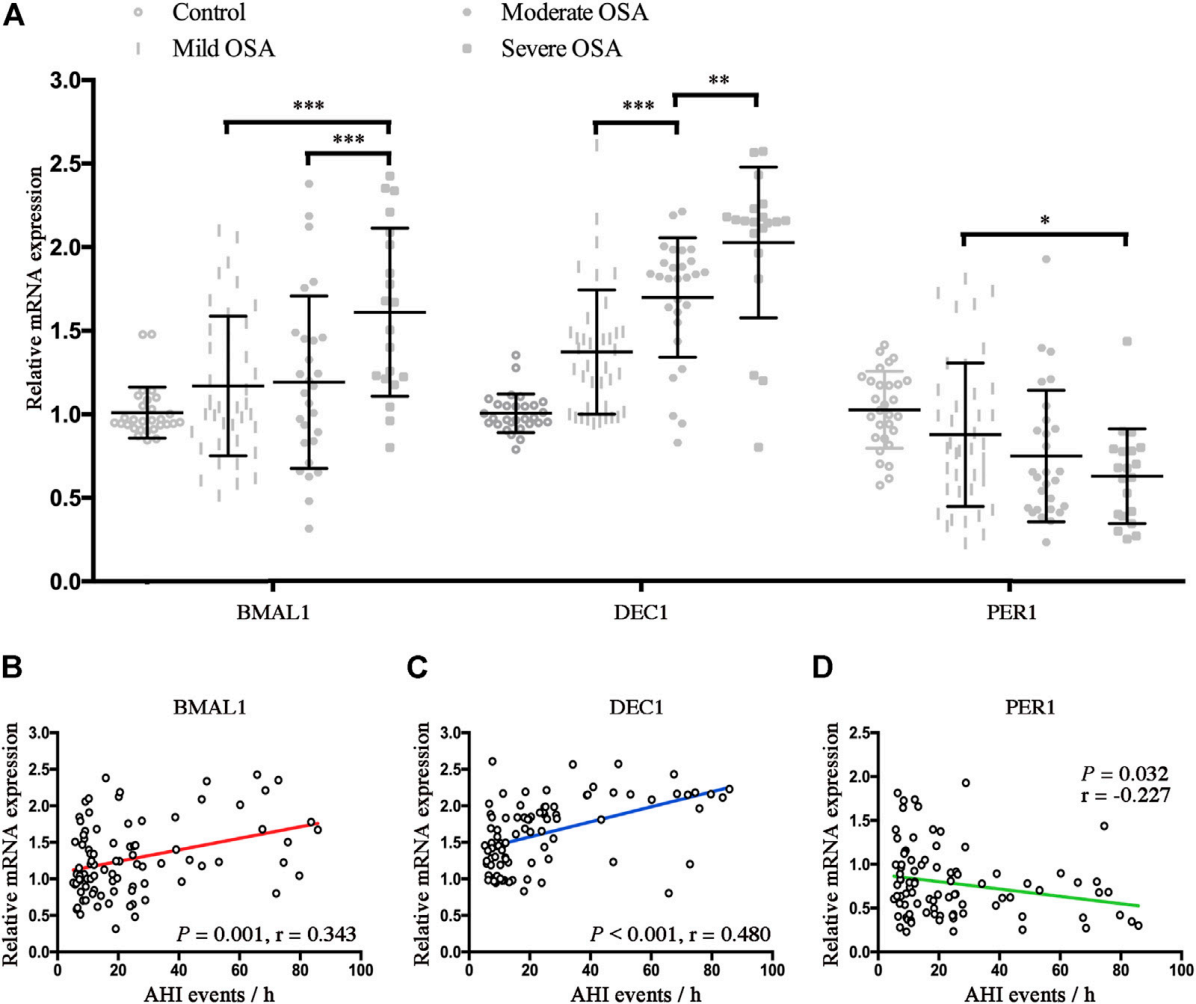
CIH is associated with the disrupted BMAL1, Dec1, and Per1 in OSA. **(A)** Expression levels of the disrupted clock genes (Bmal1, Dec1, and Per1) in the peripheral blood mononuclear cells from control and OSA subgroups (mild, moderate, and severe). **(B–D)** Linear regression between expression levels of Baml1 **(B)**, Dec1 **(C)**, Per1 **(D)**, and AHI. *, *p* < 0.05; **, *p* < 0.01; ***, *p* < 0.001.

To further investigate the effect of hypoxia on alternations of clock genes, we further compared the relative mRNA expressions of Bmal1, Dec1, and Per1 in control and OSA patients. As shown in Figure 3A, mRNA expressions of Bmal1 were significantly higher in the severe OSA patients than mRNA expressions of Bmal1 in the mild or moderate OSA patients (*p* < 0.001 for both). Similarly, mRNA expressions of Dec1 also increased with the severity of OSA (mild vs. moderate, *p* < 0.001; moderate vs. severe, *p* < 0.01). However, Per1 mRNA expression was significantly lower in the severe OSA than Per1 mRNA expression in mild OSA (*p* < 0.05). No significant differences were found for mRNA expressions of Bmal1, Dec1, and Per1 between control and mild OSA groups. We next performed the linear regression analyses to determine associations of AHI with expression levels of BMAL1, Dec1, and Per1 in OSA patients. As shown in Figures 3B,C, both Baml1 and Dec1 were positively correlated with the AHI (*r* = 0.343, *p* = 0.001 for Bmal1; *r* = 0.480 *p* < 0.001 for Dec1). However, increased AHI conferred a gentler decreasing trend for Per1 mRNA expressions in OSA (*r* = −0.227, *p* = 0.032).

### Disrupted clock genes are risk factors for the presence of MetS components in OSA

**FIGURE 4:**
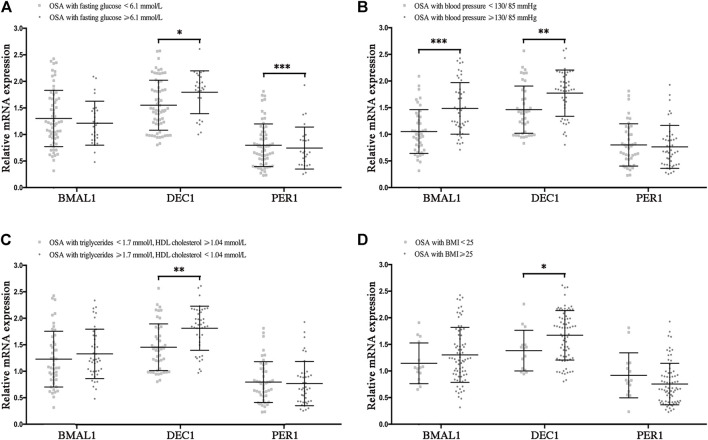
Disrupted clock genes are risk factors for the presence of MetS components in OSA. Expression levels of the disrupted clock genes (Bmal1, Dec1, and Per1) in the peripheral blood mononuclear cells from the following indicated OSA subgroups: **(A)** fasting glucose<6.1 mmol/L vs. fasting glucose ≥6.1 mmol/L; **(B)** blood pressure <130/85 mmHg vs. blood pressure ≥130/85 mmHg; **(C)** triglycerides <1.7 mmol/L and HDL cholesterol ≥1.4 mmol/L vs. triglycerides ≥1.7 mmol/L or HDL cholesterol<1.4 mmol/L; **(D)** BMI <25, BMI ≥25; *, *p* < 0.05; **, *p* < 0.01; ***, *p* < 0.001.

To determine the effect of the disrupted clocks on MetS components, we compared mRNA levels of Bmal1, Dec1, and Per1 in OSA patients with or without hyperglycemia, hypertension, hyperlipidemia, and obesity, which were considered as the major components of MetS. In OSA with hyperglycemia (fasting glucose ≥6.1 mmol/L) patients, mRNA levels of Dec1 were higher (*p* < 0.05), and those of Per1 were lower (*p* < 0.001) than those of OSA without hyperglycemia patients (fasting glucose<6.1 mmol/L). No significant differences in mRNA expressions of Bmal1 were found between OSA with and without hyperglycemia groups (Figure 4A). We also found mRNA levels of Bmal1 and Dec1 were significantly increased in the OSA with hypertension group (blood pressure ≥130/85 mmHg) compared to the OSA without hypertension group (blood pressure <130/85 mmHg, *p* < 0.001 for Bmal1 and *p* < 0.01 for Dec1), while no significant difference for Per1 was found between these two groups (Figure 4B). In addition, Dec1 mRNA expression was much higher in OSA with hyperlipidemia (triglycerides ≥1.7 mmol/L or HDL cholesterol <1.4 mmol/L, *p* < 0.01) or obesity (BMI ≥25, *p* < 0.05) comparing to that of the control groups (Figures 4C,D). Collectively, all these data indicated that OSA-disrupted expressions of Baml1, Dec1, and Per1 are involved in the pathogenesis of MetS components.

### Increased DEC1 anticipates MetS and insulin resistance in OSA

**FIGURE 5:**
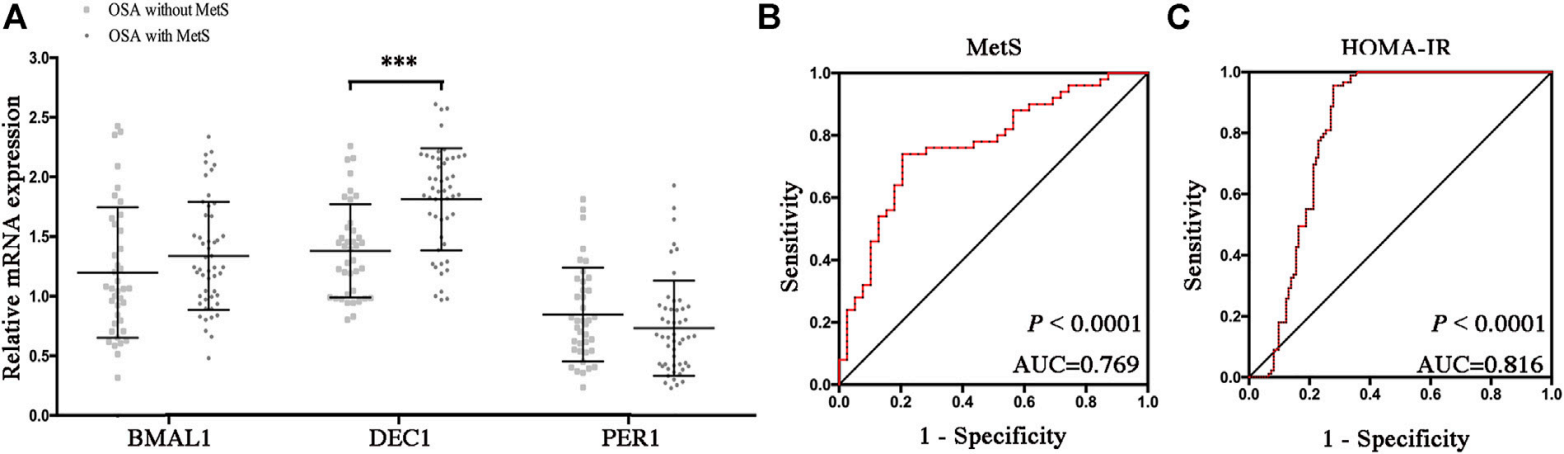
Increased DEC1 anticipates MetS and insulin resistance in OSA. **(A)** Expression levels of the disrupted clock genes (Bmal1, Dec1, and Per1) in the peripheral blood mononuclear cells between OSA-non-MetS and OSA-MetS subgroups (OSA-non-MetS vs. OSA-MetS). **(B–C)** ROC curve analyses were performed to evaluate the discriminatory power of Dec1 for MetS **(B)** and HOMA-IR **(C)**. ***, *p* < 0.001.

To determine which disrupted clock genes were involved in the presence of MetS, we further compared mRNA levels of Bmal1, Dec1, and Per1 in OSA patients with or without MetS. As shown in Figure 5A, mRNA expression levels of Dec1 were significantly increased in the OSA-MetS group compared to the OSA-non-MetS group (*p* < 0.001). However, no significant differences were found for Baml1 and Per1 between OSA-MetS and OSA-non-MetS groups. All these data suggested circadian clock Dec1, increased by the extent of CIH, may play a critical role in MetS pathogenesis.

IR has been considered as the core mechanism for developing MetS. Therefore, we performed ROC curve analyses of Dec1 (Figures 5B,C) for both MetS and IR. We observed that the areas under the curve (AUCs) for anticipation of MetS and IR were 0.769 and 0.816, respectively (*p* < 0.0001, for both). These results indicated that Dec1 expression levels in PBMCs may be used as a biomarker to predict MetS and IR in OSA patients.

### Disrupted clock genes reflect the inflammatory and oxidant status in OSA

Chronic inflammatory response, one of the major hallmarks of OSA, has been considered to potentially cause MetS (Maniaci et al., 2021). Therefore, we further detected inflammatory markers in the OSA patients and tried to reveal the correlations of these inflammatory factors with circadian clock alternations. As shown in Table 2, 12-cytokines including IL-5, INF-α, IL-2, IL-2, IN-6, IL-1β, IL-10, IL-γ, IL-8, IL-17, IL-4, and IL-12P70, and TNF-α were measured to assess the level of inflammatory responses in OSA and MetS. The results indicated that IL-5 and IL-6 were much higher in the OSA group than those in the control group (*p* = 0.037). In the OSA group, OSA-MetS patients showed higher levels of IL-6 than the non-MetS patients (*p* = 0.022). However, no significant differences were found for other cytokines in the study cohort. These data suggest IL-5 and IL-6 are involved in the development of OSA, while IL-6 may be served as a key link between OSA and MetS.

**TABLE 2:** Correlations of 12-cytokine levels with the presence of MetS in OSA patients.

Variable	Control	OSA		*P* value^a^	*P* value^b^
		Non-MetS	MetS		
**IL-5**	2.29 ± 0.96	2.77 ± 1.31	3.05 ± 1.67	**0.037**	0.395
INF-α	1.73 ± 1.29	1.30 ± 0.91	1.60 ± 1.30	0.288	0.220
IL-2	1.15 ± 0.90	1.22 ± 0.92	1.14 ± 0.78	0.874	0.665
**IL-6**	3.67 ± 2.37	7.05 ± 4.88	10.44 ± 7.93	**<0.001**	**0.022**
IL-1β	3.74 ± 4.22	3.26 ± 2.53	2.73 ± 1.69	0.188	0.239
IL-10	0.97 ± 0.31	0.90 ± 0.56	0.84 ± 0.46	0.320	0.568
IL-γ	8.20 ± 7.24	8.29 ± 3.82	7.31 ± 3.98	0.666	0.243
IL-8	2.93 ± 1.99	2.50 ± 1.06	2.71 ± 1.80	0.385	0.503
IL-17	1.65 ± 0.84	1.47 ± 1.31	1.55 ± 1.19	0.583	0.789
IL-4	1.87 ± 0.37	1.80 ± 0.43	1.75 ± 0.31	0.241	0.622
IL-12P70	1.07 ± 0.75	0.94 ± 0.66	1.16 ± 1.07	0.966	0.273
TNF-α	1.77 ± 1.88	1.74 ± 1.95	1.89 ± 1.39	0.889	0.691

These bold values mean *p* < 0.05. Data are shown as mean (standard deviation) or percentages. ^a^ ^/^ ^b^Correlation coefficients of the cytokine levels between groups.

a of control and OSA, or subgroups

b of OSA-non-MetS and OSA-MetS.

**FIGURE 6:**
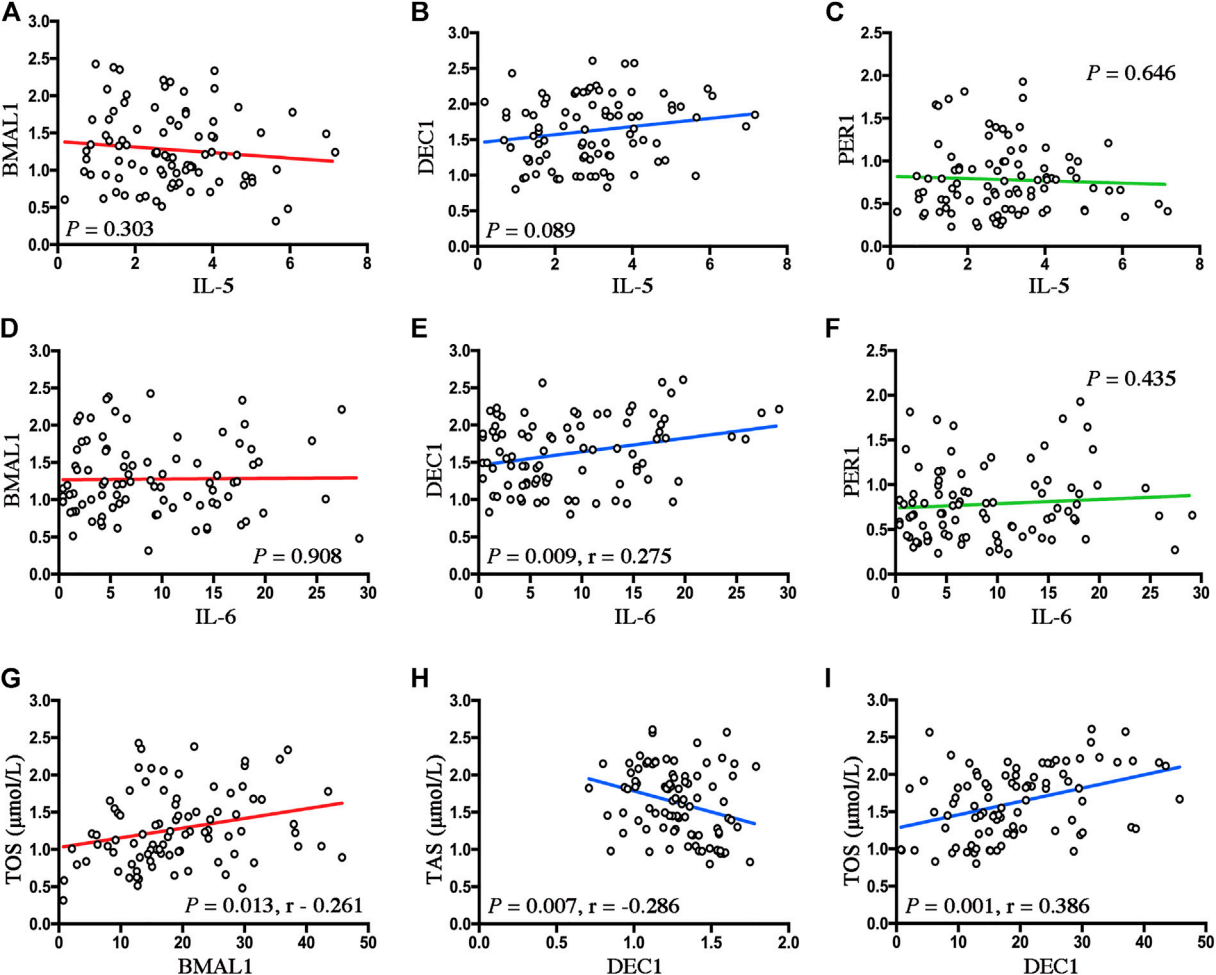
Disrupted clock genes reflect the inflammatory and oxidant status in OSA. **(A–C)** Linear regression between expression levels of Baml1 **(A)**, Dec1 **(B)**, Per1 **(C)**, and IL-5. **(D–F)** Linear regression among expression levels of Baml1 **(D)**, Dec1 **(E)**, Per1 **(F)**, and IL-6. **(G–I)** Linear regression between expression levels of Baml1 and TOS **(G)**; Dec1 and TSA **(H)**; Dec1 and TOS **(I)**.

To determine the underlying inflammatory pathways involved in the disruptions of clock genes by OSA, we also performed linear regression analyses to examine the relationship between OSA-upregulated inflammatory cytokines (IL-5 and IL-6) and disrupted clock genes (BMAL1, Dec1, and Per1) in OSA patients. As shown in Figure 6, no significant correlations between disrupted clocks (BMAL1, Dec1, and Per1) and IL-5 (Figures 6A–C) were found in the present study cohort. Although no significant correlations were observed between the disrupted clocks (BMAL1 and Per1) and IL-6, we found a positive correlation between Dec1 mRNA expressions and IL-6 levels in OSA patients (*r* = 0.275, *p* = 0.009, Figures 6D–F). These data indicate connections between Dec1 and IL-6, which regulate chronic low-grade inflammation, may be considered as an important pathological mechanism in OSA-induced MetS.

It is well known that oxidative stress is closely associated with the components of MetS, such as obesity, hypertension, hyperlipidemia, and diabetes (Martins et al., 2021). Therefore, we next explored whether these associations between OSA and oxidant status could be impacted by the disrupted circadian clocks in OSA patients. As shown in Table 3, although no significant differences for TAS were found between the control and OSA groups, TOS was significantly upregulated, and OSI was strongly downregulated in the OSA group compared to the control group (*p* < 0.001 for both). In addition, TAS and OSI were notably decreased (*p* = 0.039 and 0.024, respectively), while TOS was markedly increased (*p* < 0.001) in the OSA-MetS group compared with the OSA-non-MetS group. These data suggest that enhanced oxidative stress was involved in both OSA and MetS development.

**TABLE 3:** Correlations of oxidative indicators with the presence of MetS in OSA patients.

Variable	Control	OSA		*P* value^a^	*P* value^b^
		Non-MetS	MetS		
TAS (μmol/L)	1.37 ± 0.28	1.35 ± 0.24	1.24 ± 0.22	0.119	**0.039**
TOS (μmol/L)	5.86 ± 2.58	13.89 ± 5.78	23.34 ± 10.65	**<0.001**	**<0.001**
OSI	0.31 ± 0.22	0.17 ± 0.26	0.07 ± 0.08	**<0.001**	**0.024**

These bold values mean *p* < 0.05. Data are shown as mean (standard deviation) or percentages. ^a^ ^/^ ^b^Correlation coefficients of the cytokine levels between groups.

a of control and OSA, or subgroups.

b of OSA-non-MetS and OSA-MetS.

We next explored the correlations between circadian clock expressions and oxidative levels by performing linear regression analyses. As shown in Figures 6A,G, positive correlation between levels of Bmal1 and TOS was found in OSA patients (*r* = −0.261, *p* = 0.013). In addition, Dec1 expressions were not only negatively correlated with the TAS levels (*r* = −0.286, *p* = 0.007, Figure 6H) but also positively correlated with the TOS levels in OSA patients (*r* = 0.386, *p* = 0.001, Figure 6I). These data suggest that alternations of the clock genes are closely associated with levels of oxidative stress in OSA.

## Discussion

Epidemiologic evidence supports the interrelationships of OSA and Mets components that influence and promote each other. Recent research works showed that the biological clock was disrupted in OSA. In addition, circadian rhythms also have a profound impact on metabolic and cardiovascular disorders. Although these underlying connections between OSA and MetS were multifaceted and complicated, insulin resistance has emerged as a common pathophysiological mechanism and circadian clocks might be involved in the molecular changes. Indeed, whether which and how clock genes link OSA with Mets warrants further investigation. This study provides definitive evidence that circadian clock disruptions (Bmal1, Dec1, and Per1) are involved in the development of the MetS component. These disrupted clock genes, especially Dec1, are associated with systemic inflammation, oxidative stress, and IR. Circadian clock Dec1 can not only be used as a hypoxic index of OSA severity but also showed a predictive value for the presence of MetS in OSA patients.

Increasing incidence of Mets has been defined as a clinical feature and hallmark of OSA (Gasa et al., 2011; Murphy et al., 2017; Gaines et al., 2018; Hirotsu et al., 2018). A previous prospective cohort study has reported that OSA is an independent risk factor for incidence of MetS, but not *vice versa*. Although the presence of Mets did not lead to a growing incidence of OSA in the general population, moderate-to-severe OSA led to a 2-fold increase in the incidence of MetS, which was closely associated with nocturnal hypoxemia (Hirotsu et al., 2018). Another cross-sectional study also described the interrelationships between OSA and metabolic abnormalities, and showed definitive proof of the higher BP, and poorer lipid and glucose control that linked OSA to MetS (Gasa et al., 2011). Consistent with these studies, we also found that MetS components showed a higher prevalence among OSA patients than the non-OSA subjects (Table 1). In addition, we further evaluated levels of HOMA-IR in all participants and found that the well-known index of MetS was significantly correlated with the presence of OSA, which suggested tangled and complex associations among OSA, MetS, and IR. Indeed, mounting evidence has been pointed out with regard to insulin resistance and impaired glucose control among OSA patients, which are also linked to OSA severity and the presence of MetS (Murphy et al., 2017; Gaines et al., 2018; Mokhlesi et al., 2021). However, the specific molecular mechanism in connections of OSA-MetS has not yet been elaborated.

IR is wildly considered as the core mechanism in regulating MetS development. The predisposing effect of hypoxia on IR has been generally studied in both clinical cohorts and molecular mechanisms in the past decades (Gasa et al., 2011; Takikawa et al., 2016; Murphy et al., 2017; Thomas et al., 2017; Gaines et al., 2018; Hirotsu et al., 2018; Tang et al., 2020; Mokhlesi et al., 2021). For instance, endothelial-specific knockout of Argonaute 1, a key regulator in endothelial hypoxic response, protected from high-fat and high-sucrose induced obesity and IR in mice models (Tang et al., 2020). Another *in vivo* study also indicated that CIH-induced systemic and tissue-specific IR was independent of hypoxia-inducible factor 1 (HIF-1), but through activating the AMPK pathway. In addition, myeloid cell-HIF-1α-knockout mice improve the glucose metabolism in an anti-high-fat-diet-induced inflammatory manner (Takikawa et al., 2016). These investigations may provide some insight into how IR is activated during OSA-induced Mets. However, another possible candidate mechanism may be the dysfunction of the circadian clock network.

Cross-sectional studies have demonstrated that the circadian rhythm of clock genes was impaired in OSA (Yang et al., 2019; Gaspar et al., 2021). Yang et al. (2019) reported that oscillating reduction of Cry1 and Per3 occurred in a time-dependent manner (mainly at midnight) and served as a specific hallmark of severe OSA patients. Laetitia et al. found that OSA altered circadian rhythms of biological clocks, which could be re-established by continuous positive airway pressure (CPAP) (Gaspar et al., 2021). All these results support the hypothesis that disruption of the clock genes could be triggered under hypoxic events. Therefore, it is not surprising that the abnormal circadian rhythm of PER1 in OSA patients could be recovered by CPAP treatment (Burioka et al., 2008). Another study demonstrated that expression levels of HIF-1α were significantly upregulated and strongly associated with the altered circadian clock proteins in OSA patients (Gabryelska et al., 2020). This finding provides direct evidence for the dysregulation of circadian clocks *via* the hypoxia pathways. Indeed, some of the clock genes, such as Bmal1, Cry1/2, Dec1, and Per1, have been identified as hypoxia-responsive genes that contain HIF binding sites and play key roles in mediating oxygen adaptation (Miyazaki et al., 2002; Adamovich et al., 2017; Wu et al., 2017). Consistently, the present study confirms a similar finding that alternations of increased Baml1 and Dec1, but decreased Per1 exist in OSA patients (Figure 3). All the abovementioned observations revealed that some disrupted clocks could be not only served as an efficient hypoxic biomarker but also used for potential candidate targets in OSA treatment.

On the other hand, disrupted circadian clocks are also closely correlated with MetS components and glucose homeostasis. Recent *in vivo* studies indicate that peripheral clocks dysregulations in the liver, adipose, kidney, and smooth muscles are involved in the development of hypertension, diabetes, and obesity (Chang et al., 2018; Nakashima et al., 2018; Hou et al., 2019; Murata et al., 2020; Pati et al., 2021). In Dec1-deficient mice models, expression of Atp1b1, a negative transcriptional target of Dec1 and anti‐phase regulator of blood pressure rhythm, was upregulated in the kidney, aorta, and heart tissues, and in turn reduced blood pressure (Nakashima et al., 2018). Yu et al. (2019) have indicated that mRNA levels of BMAL1, Clock, Cry1, Cry2, and Per1 were significantly decreased and interrelated with pro-inflammatory biomarkers of IL-6 and TNF in diabetic patients. Another diabetic ob/ob mice model showed that some of the clock-controlled genes (Bmal1, Clock, Cry1, Dbp, Per1, and Per2) were substantially dysregulated in the liver and adipose tissues, which was prior to metabolic abnormalities (Ando et al., 2011). Either way, most of the studies have confirmed that the rhythm of the clock genes can be generally impaired by OSA and this disruption plays a key role in metabolic processes. It is possible that circadian clocks serve as a regulating link between OSA and metabolic dysfunction. Consistent with the above studies, we found that expressions of Bmal1, Dec1, and Per1 were not only dysregulated in OSA but also have a significant impact on the presence of MetS components, such as hyperglycemia (Dec1 and Per1, *p* < 0.05 and 0.001, respectively), hypertension (Bmal1 and Dec1, *p* < 0.001 and 0.01, respectively), hyperlipidemia (Dec1, *p* < 0.01), and obesity (Dec1, *p* < 0.05). To the best of our knowledge, the present study is the first to explore roles of clock genes in connections between OSA and MetS.

Oxidative stress and systemic inflammation are known major hallmarks of OSA and involve in a spectrum of cardiovascular and metabolic pathophysiological progression, as reviewed by Maniaci et al. (2021). Recently, Yamauchi et al. (2005) found that urinary 8-hydroxy-2-deoxyguanosine (8-OHdG), an oxidative index *in vivo*, was significantly increased in severe OSA patients and closely correlated with the hypoxia-related indicators following PSG. Hypoxia conditions impair the oxidant–antioxidant balance and break the redox homeostasis toward the oxidative side (Bozkurt et al., 2015). Therefore, it is clear that with the severity of intermittent hypoxia, a trend of a progressive decrease of TAS, and a significant increase of thiobarbituric acid-reacting substances (TBARS, an index of lipid peroxides) were found from non-to-mild, moderate, and severe OSA patients (Cofta et al., 2019). Consistent with these studies, we found the oxidative indicator TOS and the anti-oxidative indicator OSI were significantly correlated between the control and OSA groups. Furthermore, levels of TAS, TOS, and OSI were all significantly correlated between the OSA-non-MetS and OSA-MetS groups (Table 3). Collectively, all these results indicated that oxidative stress serves as another important hallmark and link in OSA with MetS.

In view of the increased ROS production by CIH-reoxygenation cycles, another critical consequence is presented with inflammation. Likewise, enhanced systemic inflammation had been repeatedly reported in the pathogenesis of both OSA and MetS. Actually, other previous and present studies have implicated the upregulation of pro-inflammatory cytokines in the progression of OSA and its complications through hypoxia-responsive and insulin signaling (Kritikou et al., 2014; Murphy et al., 2017). For instance, the findings of Murphy et al. (2017) demonstrated that intermittent hypoxia promoted the inflammation of visceral adipose tissue *via* M1 macrophage polarization and reduced the glucose uptake in 3T3-L1 adipocytes in an insulin-signaling pathway. Another study further confirmed OSA was closely associated with IR and inflammation even independent of obesity. They found apnoeic males showed higher hsCRP, IL-6, leptin, and insulin resistance than controls, while no significant influence can be observed in these biomarkers after CPAP treatment (Kritikou et al., 2014). In our study, we found that levels of IL-5 and IL-6 in OSA subjects were much higher than those of the controls, while IL-6 levels showed significant correlations between OSA-non-MetS and OSA-MetS groups (Table 2). Likewise, increased evidence has also been pointed out the role of IL-6, a biomarker of OSA, in mediating connections among OSA, IR or Mets (Kim et al., 2009; Maeder et al., 2015; Akbari and Hassan-Zadeh, 2018; Motamedi et al., 2018; Ko et al., 2019; Imani et al., 2020; Campos-Rodriguez et al., 2021), Although the molecular mechanisms are not clear, our findings indicate that these clocks / oxidative stress / inflammation / IR interactions play a key role in the linkage between OSA and MetS. Among the disrupted circadian clocks, Dec1 should be supposed to involve in these interactions, in view of its correlations with HOMA-IR, presence of Mets, TAS, TOS, and IL-6 (Figures 5, 6). Indeed, several studies provided evidence for this notion. First, among all the OSA-disrupted clock genes, Dec1 shows the most prominent change and the most specific OSA effect in OSA patients during the morning (Gaspar et al., 2021). Second, as a hypoxia-responsive and stress-associated protein, others and we have been found that Dec1 transcriptionally suppresses another transcriptional factor peroxisome proliferative activated receptor-γ (PPARγ), a key mediator in regulating insulin sensitivity (Yun et al., 2002; Li et al., 2021). Third, interactions of both Dec1/PPARγ and Dec1/IL-6 occur under inflammatory or oxidative burden and involved in cellular energy metabolism (Yun et al., 2002; Ning et al., 2017; Noshiro et al., 2020; Luan et al., 2021). Fourth, Dec1 has been reported to modulate the process of occurrence and development of MetS components, including hypertension, diabetes, and obesity (Yun et al., 2002; Yamada et al., 2003; Chen et al., 2015; Nakashima et al., 2018; Noshiro et al., 2018).

The study has some limitations: 1) the lack of a CPAP interventional arm makes it difficult to infer the role of hypoxia intervention in predicting better MetS outcomes in OSA subjects. Further data collection (e.g., clock gene expressions after CPAP treatment in OSA patients) is needed to make this more explicit. 2) Ethically speaking, we can only obtain the morning fasting blood samples of the participants. This precluded us from clarifying the expression difference of tissue-specific clock genes. In addition, circadian oscillation alternations of the clock genes were also unable to assay all day long. 3) Blood samples are limited in size; this study only detected part of the clock genes. There might have been other clock genes that impact MetS development in OSA patients.

In summary, these data suggest a new understanding of circadian clock disruptions in OSA patients and how these genes impact Mets development. Both oxidative stress and systemic inflammation are associated with specific clock gene alternations, and this might cause IR and endothelial dysfunction. Circadian clock disruptions might be the core link of the potential mechanism of OSA promoting cardiovascular metabolic diseases. Furthermore, Bmal1, Dec1, and Per1 may be independent predictors and potential therapeutic targets in OSA patients with MetS.

## Data Availability

The original contribution presented in the study are included in the article/Supplementary Material; further inquiries can be directed to the corresponding authors.
