# Supplementary material for: Circadian clock disruptions link oxidative stress and systemic inflammation to metabolic syndrome in obstructive sleep apnea patients
Source: Front Physiol. 2022 Aug 29;13:932596. doi: 10.3389/fphys.2022.932596 (PMC9466597; doi:10.3389/fphys.2022.932596)
Supplement: Supplementary file 1 [file Table1.docx]

**Supplementary Table 1:** Primer sequences for the qRT-PCR

| Gene | Primer sequences (F: Forward sequence; R: Reverse sequence) |
| --- | --- |
| *Bmal1* | F: AGATGAATTGGCTTCTTTGG |
|  | R: TGGCACCTCTTAATGTTTTC |
| *Clock* | F: ACTACAAGACGAAAACGTAG |
|  | R: CATCTCTGTCAACAATCG AG |
| *Cry1* | F: CCCAATGGAGACTATATCAGG |
|  | R: ACCTTTTGGATACCTTCTGG |
| *Cry2* | F: CTTTCTTCCAGCAGTTCTTC |
|  | R: CTCATAGATGTATCGAGAGGG |
| *CSNK1ε* | F: CTCCGAATTCTCAACATACC |
|  | R: AATTTCAGCATGTCCCAGTC |
| *Dec1* | F: CAAGTGTACAAGTCAAGACG |
|  | R: GTCTCTTTTTCTCGATGAGC |
| *NR1D1* | F: CTCAAAGAATGTTCTGCTGG |
|  | R: GTGAAGCTCATGGAGAAATC |
| *Per1* | F: ACACTTCAGAACCAGGATAC |
|  | R: AGTGGAACCATAGAAGACTC |
| *Per2* | F: GCCAATGAAGAGTATTACCAG |
|  | R: ATTCTTCACAATGTGCTCAG |
| *Per3* | F: AAGTTTTGAAGTATGCAGG |
|  | R: TCCAGTATGATGTAGTCTCC |
| *Gapdh* | F: GCCCAATACGACCAAATCC |
|  | R: ACAGTCAGCCGCATCTTC |
